# Drug-specific Treg cells are induced during desensitization procedure for rituximab and tocilizumab in patients with anaphylaxis

**DOI:** 10.1038/s41598-021-91851-7

**Published:** 2021-06-15

**Authors:** Alessandra Vultaggio, Francesca Nencini, Susanna Bormioli, Elena Silvestri, Laura Dies, Emanuele Vivarelli, Enrico Maggi, Andrea Matucci

**Affiliations:** 1 Immunoallergology Unit, Department of Medicine and Geriatrics, Careggi University Hospital, Florence, Italy; 2 Centre for Research, Transfer and High Education DENOTHE, Department of Experimental and Clinical Medicine, University of Florence, Florence, Italy; 3 Interdisciplinary Internal Medicine Unit, Neuroskeletal Department and Sense Organs, Careggi University Hospital, Florence, Italy; 4 Translational Immunology Unit, Immunology Area, Pediatric Hospital Bambino Gesù, I.R.C.C.S., Rome, Italy

**Keywords:** Lymphocytes, Biologics

## Abstract

Biologic agents (BA) are able to induce an adaptive immune response in a proportion of exposed patients with the onset of anti-drug antibodies (ADA), which are usually responsible for hypersensitivity reactions (HR). Drug desensitization (DD) for BA allows transient clinical tolerance to the drug in reactive patients. The paper aimed to analyse the modification of drug-specific immune responses along DD in two patients with previous ADA-mediated HR (anaphylaxis) to rituximab and tocilizumab. The in vivo and in vitro assays of humoral and cellular response to drugs were carried out in a longitudinal manner throughout the DD cycles. We observed a progressive decrease of the pre-procedure ADA titer with negativization during the DD cycles in both patients. The monitoring of the drug-specific effector cell response showed the decrease in the BA-induced proliferation, while T cell response to unrelated antigens resulted unmodified along the DD cycles. Lastly, the increase of circulating drug-specific Treg cells mainly producing IL-35 were shown during the DD treatment. This study provides evidence that DD treatment to two BA inhibits humoral and cellular anti-drug response by increasing regulatory T cells and cytokines in an antigen-restricted manner. These modifications could contribute to the safety of the procedure.

## Introduction

Drug desensitization (DD) is a procedure that allows for temporary clinical tolerance to a drug, by administering gradually increasing small doses to complete the total therapeutic dose of the drug^1–3^. Drug desensitization is increasingly applied^4–6^ for patients with hypersensitivity reactions (HRs) to biologic agents (BAs). These engineered molecules are usually immunogenic and are able to induce cellular and humoral immune responses in a proportion of patients with the production of anti-drug antibodies (ADAs) that are involved in the majority of adverse events^5,7^. Different ADA isotypes have been observed during biological treatment: IgG, mostly of IgG1 and IgG4 subclasses, but also IgE, IgM, and IgA^8,9^. Clinical experience shows that DD is effective in IgE and non-IgE mediated HRs^10^.

The mechanism(s) underlying this effect is poorly understood. In studies performed in animal models, it has been shown that DD inhibits the phosphorylation of intracellular signaling in mast cells (MC), which prevents the mediator release^11,12^. Other authors have also shown the inhibition of actin polymerization leading to a higher stability of intracellular granules of MC. Mast cells desensitized to one antigen are responsive to a second non desensitizing antigen, likely due to a compartmentalization and antigen-specific intracellular process^2,13,14^. Furthermore, very poor data have been produced on a possible involvement of adaptive response to the drug and its regulation in DD^15^. In a previous paper, we have shown that prolonged treatment with infliximab, a TNF-a blocker, induced IL-10-producing memory T cells that can prevent ADA development in exposed patients^16–18^. We also took advantage from results from a patient undergoing DD to infliximab who developed an initial up-regulation of drug-specific T cell response during the procedure followed by the increase of drug-specific T regulatory cells^19^.

The present study has been addressed to evaluate the adaptive response to BA other than infliximab during the DD procedure in order to establish if the improvement of Treg cells is a general phenomenon associated with this type of treatment. For this purpose, we analysed antigen-specific immune responses during DD procedure in two selected patients with previous HR to a first-line BA. The in vivo and in vitro studies on humoral and cellular responses to the drugs (rituximab—RTX- and tocilizumab—TCZ-) were carried out in a longitudinal manner throughout the DD cycles.

## Methods

### Patients

A panel of 10 patients suffering from autoimmune disorders or lymphomas, showing HR during the starting infusions with RTX (anti-CD20 mAb) or TCZ (anti-IL-6R mAb) and undergoing to DD procedure, has been selected. We focused the study on two patients who showed the following features: each patient suffered a hypersensitivity reaction during treatment with a first-line monoclonal antibody and resulted positive for IgG- and IgE ADA. Only patient #1 was tested cutaneously with the culprit drug, as patient #2 had extremely thin and delicate skin, which rendered impossible an allergologic evaluation.

Rituximab treated patient (patient #1) was a 69-year-old male with splenic marginal B lymphoma treated with first line therapy with bendamustine plus rituximab in the oncology ward. During his first infusion, the patient presented with chills and generalized tremors when the infusion rate was increased from 100 to 150 cc/h. The infusion was interrupted for 30 min and then restarted at the tolerated infusion rate of 100 cc/h until full dose was reached. One month later, at the beginning of the second RTX administration, the patient presented a severe anaphylactic reaction characterized by thoracic oppression, hypotension and desaturation. At this time, no tryptase levels were acquired and the patient was transferred to the emergency unit where he was slowly stabilized. Two months later the patient was referred to our service for allergological evaluation: non isotype specific anti-RTX antibodies resulted positive at high titer (4729 AU/ml). Skin Prick Test (SPT) were positive with histamine, grass pollen (LoPharma, Milano) and RTX at 1:1. Taking into account the clinical history with an immediate severe adverse reaction and in vitro and in vivo results, we decided to proceed with a 4-bag 16-step DD protocol. Patient #1 concluded 5 DD cycles from July to November 2017. Skin testing was repeated after the first desensitization, before, and after each following procedure. He returned after protocol completion for follow-up visits to repeat SPT and blood sampling to assess immune response to the drug.

Tocilizumab-treated patient (patient #2) was a 78-year-old male, with a diagnosis of giant cell arteritis (GCA), in whom, despite prolonged therapy with corticosteroids, occlusion of the central retinal artery was documented. The patient promptly began treatment with TCZ (300 mg) from October to December 2017 with no adverse reactions until December 2017 when treatment was interrupted for haematologic investigations (BM osteo-biopsy that confirmed JAK2 + chronic myeloproliferative syndrome). After a suspension of 5 months treatment was restarted, and during the second infusion, the patient presented with pruritus of hands and feet, promptly treated with anti-H1. The patient resulted positive for non isotype specific anti-TCZ antibodies (73 ng/ml). Given patient history, the unresponsiveness to corticosteroid therapy, and positivity of anti-TCZ ADA it was decided to proceed with a 4-bag 16-step DD protocol. Patient #2 began DD treatment on September 2018 and completed the 5 DD procedures once monthly.

This study was carried out in accordance with the recommendations of Internal Ethical Committee of Azienda Ospedaliera Universitaria Careggi (2012/0035982) with written informed consent from all subjects. All experimental protocols were approved by Internal Ethical Committee of Azienda Ospedaliera Universitaria Careggi.

All experiments were performed in accordance with relevant guidelines and regulations and adhered to the tenets of the Declaration of Helsinki and followed good clinical practice guidelines.

### Desensitization protocol

Venous blood was sampled at the following time intervals: prior to medication along with a sample for evaluating tryptase levels at T0, and after each step/bag completion. Tryptase levels were re-sampled in case of adverse reactions. For patient#1 only, samples were taken after the treatment completion at the follow-up visits. Each patient underwent ECG monitoring prior to each procedure, and remained hooked up to a vital-signs monitor in order to acquire all vital signs before and after each increase in rate infusion. The prophylactic therapeutic protocol prior to beginning of each procedure was applied, as described^19,20^. Any medication that could negatively impact life-saving procedures was withheld. An emergency cart was prepared with medications as described^21^. The schedule of each DD cycle is summarized in the Table E1.

### Reagents

Low-endotoxin RPMI 1640 medium (VLE-RPMI 1640, Biochrom AG, Germany) was supplemented with 2 mM l-glutamine, 2 mM 2-mercaptoethanol, 100 U/ml penicillin and 100 µg/ml streptomycin, 1% non-essential amino acids, 1% sodium pyruvate (all from Sigma Chemical Co, Milan, Italy) (complete medium). Human serum AB was purchased from Euroclone (Milan, Italy). Phorbol 12-myristate 13-acetate (PMA) and ionomycin (I) were purchased from Sigma-Aldrich (Milan, Italy).

Streptokinase was purchased from Behring (L’Aquila, Italy) and recombinant Phl p 5 was purchased from Biomay AG (Vienna, Austria). Anti-MHC class II antibody and isotype control were purchased from Becton–Dickinson (Mountain View, CA, USA). Antibodies and isotype controls for cytometric analysis were purchased from Miltenyi Biotec (Bologna, Italia).

### ADA detection and drug measurement

The ADA status of patients and drug serum levels was evaluated by using a commercially available ELISA kit (Promonitor, Progenika Biopharma, Bizkaia, Spain for non-isotype specific anti-rituximab antibodies and rituximab; Theradiag, MARNE LA VALLEE CX2, France for non-isotype specific anti-tocilizumab antibodies and tocilizumab), according to the manufacturer’s instructions.

Limit of detection for anti-tocilizumab antibodies was 5 ng/ml and for tocilizumab 1 µg/ml.

Limit of detection for anti-rituximab antibodies was 75 AU/ml and for rituximab 0.75 µg/ml.

### Lymphocyte proliferation test (LTT)

PBMCs from patients were isolated with Ficoll-Paque, and 2 × 10^5^ cells were cultured for 5 days in complete medium and 5% heat-inactivated human serum AB in 96-well round-bottomed microwell plates with or without RTX (50 μg/ml) and TCZ (50–25-12.5 μg/well). For the assessment of drug specific T cell response, PBMCs of patient 2# were cultured in medium alone or TCZ with or without anti-major histocompatibility complex (MHC) class II (5 μg/ml) for 5 days.

Sixteen hours before harvesting, 0.5 µCi of Tritiated Thymidine (PerkinElmer, Boston, USA) was added to each well, and radionuclide uptake was measured by scintillation counting (MicroBeta TriLux PerkinElmer, Boston, USA). Mitogenic index (ratio between mean value of counts per minute—c.p.m.—of samples and medium alone, MI) ≥ 2 was considered positive as described^17^.

### Real-time PCR

For evaluation of mRNA expression (IFN-γ, IL-13, IL-17, IL-10, IL-12A, EBI-3 and Foxp3), 1 × 10^6^ cells were cultured for 12 h in complete medium and 5% heat-inactivated human serum AB in 48-well round-bottomed microwell plates with or without RTX or TCZ (50 μg/ml).

RNA was extracted with the RNeasy mini kit (Qiagen, Milan, Italy), and cDNA was transcribed using a reverse transcription kit (Applied Biosystems, Warrington, UK) according to the manufacturer’s instructions. The real-time PCR reactions were performed in multiple replicates and run on an ABI PRISM 7700 Sequence Detector (Applied Biosystems, Warrington, UK) with predesigned TaqMan Gene Expression Assays and reagents (Applied Biosystems, Warrington, UK), according to the manufacturer’s instructions as described^16,17^.

mRNA ratios (between the value of cytokine gene expression produced in response to the drugs and the value of cytokine gene expression produced in response to medium alone) ≥ 2 were considered as positive.

### ELISPOT test

PBMCs were seeded at 2 M/ml with TCZ (50 µg/ml) in complete medium supplemented with 5% SAB, 50 IU/ml IL-2 (Miltenyi Biotec) and 2.5 µg/ml anti-CD28 (Miltenyi Biotec). The culture medium was changed every 2–3 days. Cells were harvested at day 10 and incubated with drug (50 µg/ml) and RPMI 1640 medium supplemented with 5% SAB at 37 °C in precoated 96-well PVDF bottom plate (Diaclone SAS, 188 Biotech, France). After 24 h incubation, supernatants were collected, and IFN-γ secretion was detected, according to the manufacturer’s description. Spots were counted with a computer-assisted video image analyser (AID, Strassberg, Germany).

### ELISA assays

The LTT and Elispot culture supernatants were assayed for adaptive and regulatory cytokines with commercially available ELISA kits (from R&D Systems, Minneapolis, MN for IFN-γ, IL-13, IL-17A, from Affymetrix eBioscience, Vienna, Austria for IL-10 and *CUSABIO* Biotech CO. LTD for IL-35), as described^17^.

### Cytometric analysis

1 M/ml of PBMC were collected and stained with a panel of surface markers: CD3-VioGreen, CD25-PE, CD4-APC-Vio770, and with an intracellular antibody directed against Foxp3. The cells were then analyzed by “BD FACSCanto II” fluorocytometer (Becton–Dickinson, Franklin Lakes, NJ USA) and BD FACSDiva software as described^16^ Antibodies and isotype controls were purchased from Miltenyi Biotec (Bologna, Italia) (Table E2).

### Statistical analysis

The results are presented as mean values ± SEM. The statistical analyses were performed using a two-tailed Student t test. p < 0.05 was considered statistically significant.

## Results

### Patients

This longitudinal study was addressed to evaluate the adaptive response towards two therapeutic agents targeting cell surface molecules and having similar treatment schedules (as RTX and TCZ) during the DS procedure in two patients who had previously developed a severe HR to the same drugs. They suffered from non-Hodgkin lymphoma (patient #1) and Horton’s Arteritis (patient #2) and were treated with infusive RTX and TCZ, respectively.

After 4 weeks from the HR, the patients were restarted the previous therapy by using a 16-step  DD protocol with a cumulative dose/cycle of 675 mg of RTX and 600 mg of TCZ, respectively. Intravenous DD cycles were performed monthly at d0, d30, d60, d90, d120 according to the induction/maintenance procedure of the drugs.

Before DD procedure both patients showed ADA detectable to culprit drugs in the serum and the sensitization to an environmental allergen (Phleum pratensis – Phl p5, in patient #1).

### Clinical outcomes and skin testing during the DD procedure

No HR were developed by the two patients during the DD procedure. The patient #1 displayed mild reactions during the first two DD cycles with flushing and localized urticaria, which regressed with the temporary suspension of the infusion and the subsequent recovery at a reduced infusion speed. Of note, the following DD cycles were free of adverse reactions and patients completed the administration of planned doses for each cycle. This confirms the safety and efficacy of the procedure.

It was not possible to proceed with drug-specific skin testing (both SPT and intradermal test, IDT) in patient #2, as the patient’s skin presented as extremely frail with a tendency to haemorrhage subcutaneously, likely due to both age and concomitant previous corticosteroid therapy.

**Figure 1:**
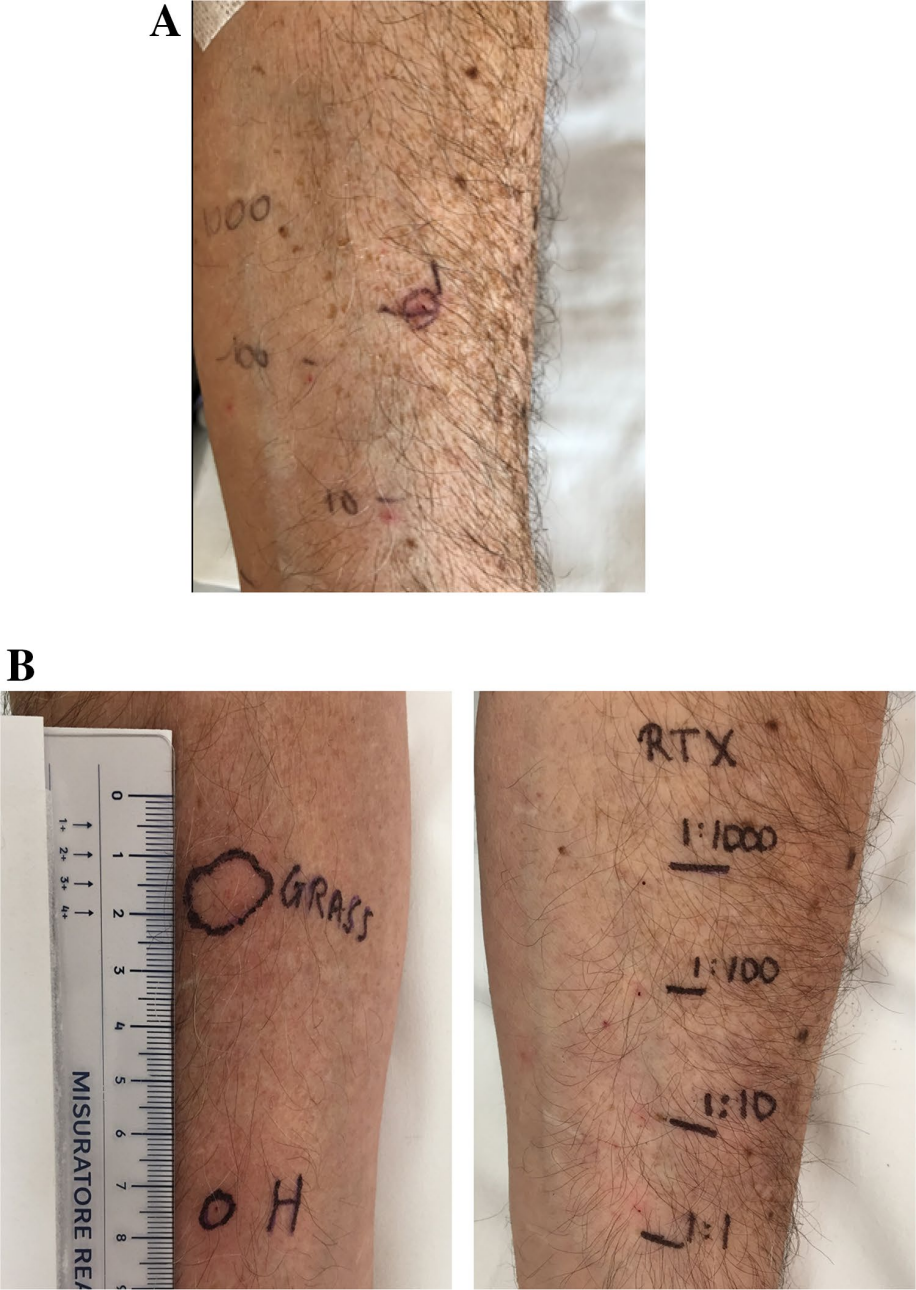
Skin testing for rituximab (IDT) before DD (**A**) and for rituximab (IDT), grass pollen extract and histamine (SPT) at the follow-up visit, 6 months after the last drug desensitization (**B**) in patient #1. *H* histamine, *IDT* intradermal test, *SPT* skin prick test, *RTX* rituximab.

Patient #1 underwent skin testing before and after each DD cycle. Prior to procedure 1, the patient tested positive for pollen and drug SPT (Fig. 1A and data not shown). At the end of the first cycle, the patient still tested positive for pollen SPT, but negative for drug (IDT 1:10). Skin testing were positive before the following DD cycle but negativized prior to the third and the subsequent cycles (Table 1).

**Table 1:** Skin testing for rituximab and grass pollen across desensitization cycles in patient #1.

	Pre 1° DD	Post 1° DD	Pre 2° DD	Post 2° DD	Pre 3–5° DD	Post 3–5° DD
RTX IDT 1 mg/ml	++	Neg	++	Neg	Neg	Neg
Grass pollen SPT	+++	+++	+++	+++	+++	+++
Positive control (histamine)	+++	+++	+++	+++	+++	+++

*DD* drug desensitization, *IDT* intradermal test, *RTX* rituximab, *SPT* skin prick test.

At the follow-up visits after 3 and 6 months post conclusion of DD cycles, the patient showed a positive reaction for histamine and grass pollen extract and persistent skin negativity for RTX IDT (Fig. 1 and data not shown).

### DD modifies humoral response to the drug

**Figure 2:**
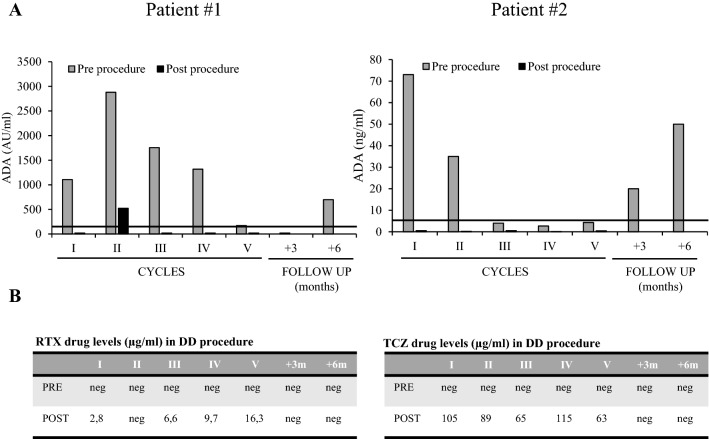
Monitoring of serum ADA (**A**) and drug levels (**B**) pre- and post-procedure during DD cycles. *ADA* anti-drug antibodies.

Serum non isotype specific ADA were evaluated immediately before and after each DD cycle. ADA were undetectable in the serum soon after each DD procedure due to drug interference in ADA assay and the formation of drug-ADA immunocomplexes and, conversely, free drugs were usually detectable in the serum. Over time in patient #1, after the increase before the second cycle, we observed a progressive decrease of the pre-procedure ADA titer with negativization before the 5th cycle. The absence of circulating RTX at trough level rules out any false negative results for ADA detection. In this patient serum ADA were still negative at the 3 months follow-up visit, while they were again later detectable at low levels (Fig. 2A,B left panel). Similarly, in patient #2 we observed a slight initial increase followed by a strong decrease with negativization of TCZ-specific ADA before the 3^rd^ and subsequent DD cycles. At both 3 and 6 months follow-up visits ADA were again detectable (Fig. 2A,B right panel).

### DD modifies cellular response to the drug

**Figure 3:**
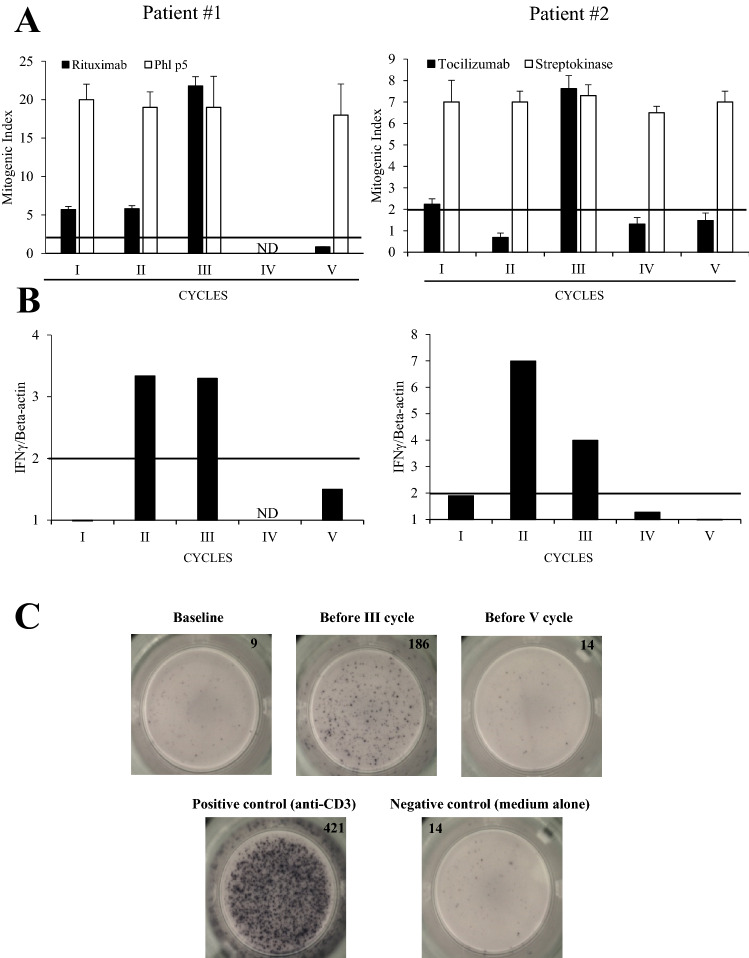
(**A**) Desensitization effects on PBMC proliferation to drug and Phl p5 allergen or streptokinase, evaluated by 3^H^ uptake. (**B**) IFN-γ mRNA expression in drug-stimulated PBMC at different desensitization cycles. (**C**) IFN-γ-producing T cells upon tocilizumab-specific stimulation on ELISPOT assay. *IFN* interferon, *PBMC* peripheral blood mononuclear cells, *Phl p5* Phleum pratensis 5.

The proliferative response of patients’ PBMC in response to the drug was monitored over time. In patient #1, the MI in response to RTX was positive prior to treatment, increased until the 3rd procedure, and became negative prior to the 5th procedure. The proliferation to grass (Phl p5) pollen simultaneously performed, was positive regardless of procedure (Fig. 3A left panel).

Similarly, in patient #2, the MI in response to TCZ dose curve was positive at the first cycle, increased at the 3rd procedure and became negative from the 4th cycle on. The proliferation to a recall antigen as streptokinase, gave positive results regardless of procedures (Fig. 3A right panel, Figure E1).

We also evaluated the adaptive cytokines (IFN-γ, IL-13, IL-17A) mRNA expression on drug-stimulated PBMC. In patient #1, IFN-γ mRNA was negative prior to treatment, became detectable before the subsequent procedures and negative from the 4th cycle on (Fig. 3B left panel). Similarly in patient #2, IFN-γ mRNA was negative before DD procedure, became detectable before the subsequent three procedures and negative at the 5th cycle (Fig. 3B right panel). Accordingly, the number of IFN-γ-producing cells evaluated by ELISPOT assay, was very low at baseline, peaked at the 3rd DD cycle and decreased later (Fig. 3C). Both IL-13 and IL-17 mRNA were undetectable in both patients throughout the DD procedure (data not shown).

### DD expands drug-specific Treg cells producing regulatory cytokines

**Figure 4:**
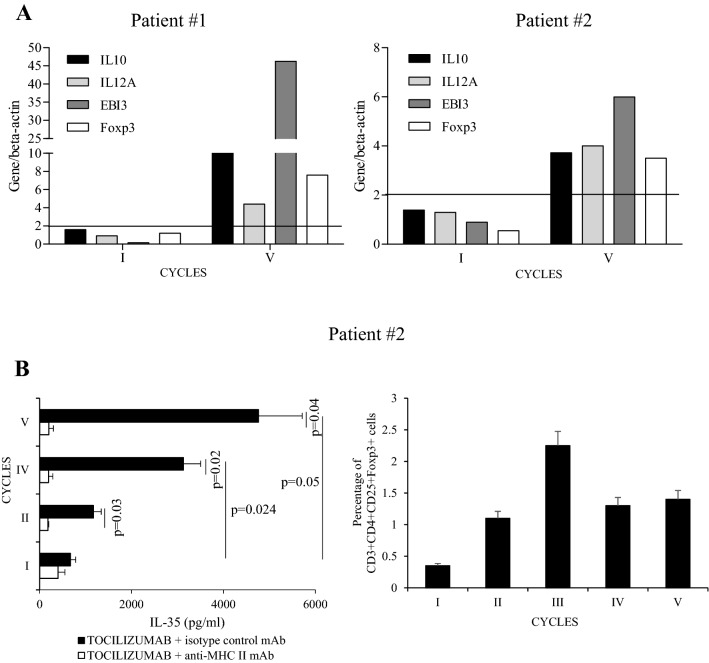
(**A**) mRNA expression of regulatory molecules in drug-stimulated PBMC. (**B**) IL-35 production by tocilizumab-specific PBMC, restricted to MHC class II elements (left panel); FACS analysis of CD25 + Foxp3 + (CD3 + CD4 + gated) cells during the desensitization procedure (right panel). *FACS* fluorescence-activated cell sorting, *IL* interleukin, *MHC* major histocompatibility complex, *PBMC* peripheral blood mononuclear cells. FACS analysis was performed by “BD FACSCanto II” fluorocytometer and BD FACSDiva software v 6.1.3 (https://www.bdbiosciences.com/en-us/instruments/research-instruments/research-software/flow-cytometry-acquisition/facsdiva-software).

Regulatory mechanisms were evaluated by mRNA expression of regulatory cytokines (IL-10 and IL-35) in drug-stimulated PBMC. In both patients we observed an increase in IL-10 and EBI3/IL-12A mRNA expression at the last cycle (Fig. 4A). In patient #2, besides evaluating the mRNA regulatory profile (Fig. 4A right panel), we had the opportunity to assess IL-35 production by PBMC upon in vitro stimulation with TCZ and we showed a significant IL-35 production in an MHC class II restricted manner. Over DD cycles, a progressive increase of IL-35 was also observed (Fig. 4B left panel). Accordingly, an increase of CD3 + CD4 + CD25 + Foxp3 + T cells was seen (Fig. 4B right panel).

## Discussion

This study investigates how immunological responses change during DD procedures for BA. We provide evidence that DD deeply modulates both humoral and cellular BA-specific immune response involving regulatory mechanisms.

The first important finding of the study concerns the decrease of ADA levels. Their longitudinal analysis highlights a transient increase in antigen-specific humoral response during the first cycles, which then convert to a sustained inhibition throughout the remaining protocol. ADA levels constantly decrease, until becoming undetectable; these values were not skewed by blood drug levels, and could indicate the beginning of immune modulation from the third procedure onwards. The monitoring of ADA showed that ADA tend to turn again positive a few months after the end of DD protocols.

We cannot exclude that part of the ADA reduction observed during the procedure could be attributed to the intrinsic activity of both RTX and TCZ on the B cell biology. RTX identifies B lymphocytes as targets causing an abrupt reduction in circulation during treatment, followed by an increase in their numbers after 6–10 months from drug interruption. However, the cells responsible for producing antibodies, plasma cells, do not express surface CD20, and therefore they are not depleted during treatment with RTX. They can, however, respond to regulatory signals^21,22^. TCZ suppresses immunoglobulin production in B cells likely through inhibition of IL-6-mediated signalling to B cells and plasma cells^23^.

Overall, this data regarding humoral anti-drug response, was supported by the study of the specific cellular response. T cells and their cytokines during DD have been examined in a few studies until now. These studies are focused on aspirin DD and provide limited and controversial information, not allowing any significant understanding of the cellular immune mechanisms operating during DD^24^. In our study, we monitor the drug-specific effector cell response, showing the decrease in the BA-induced proliferation. This data suggests that DD may be able to down-regulate the adaptive immune cell response against the drug. More importantly, the modulation of cellular response operated by DD was antigen-specific, thus representing one of the main point of our study. It is important to note that the modulation of humoral anti-drug response is a time-limited phenomenon.

It seems evident that an activation of the effector response towards the BA occurs in the very early phases of DD procedure, as suggested by that the initial increase of ADA levels and drug-induced proliferation. The increase of the effector response built against the drug in the initial phases and its regulation towards the end of the protocol are compatible with what we currently know about the adaptive immune response against exogenous antigens. However, we cannot rule out that the activation observed during the first DD cycles are partially due to the drug administration (pre-DD) that elicited HRs in both patients.

In patient #1 the unique situation of a sensitization to pollen gave us the opportunity to compare in vivo results of skin testing for grass and culprit drug (RTX). At the end of each DD cycle, the patient tested negative for drug, and positive for grass pollen, indicating an antigen-specific tuning off process of mast cells. This in vivo data agree with some already published results based on in vivo ad in vitro models showing that the exposure of mast cells to small incremental antigen doses deactivate the cells, preventing mediator release in an antigen-specific fashion^11,25^. Moreover, while the patient tested positive for the skin tests pre-procedures at the 1st and 2nd cycle, he then tested negative pre-procedure 3, and for the following procedures after that. This seems to be in accordance also with the decrease of non-isotype specific ADA observed from the third procedure onwards.

The analysis of regulatory mechanisms that could be involved in successful DD, complete the evaluation of the potential immunological modifications induced in desensitized patients. mRNA expression for regulatory cytokines showed a similar trend to the proliferative response, along with a parallelism to the regulatory mechanisms for both the humoral and cellular anti-drug response. Specifically, the increase of regulatory cytokines coincides with ADA decrease, pre-procedure skin testing negativization and impairment of T cell response, thus indicating a generalized cellular and humoral regulation.

The induction of IL-10 has been already described in successful DD^19,26,27^, while the involvement of IL-35 in regulation induced by DD is an emerging finding. Furthermore, we showed, in an MHC class II-restricted system, that IL-35 is produced by T cells going through a progressive increase during the repeated DD cycles. On the other hand, we cannot rule out that IL-10 can be released by lytic B cells (in the case of RTX-treated patient) or produced by regulatory B cells (Breg). Of note, the upregulation of IL-35 was paralleled by the Foxp3 mRNA expression, thus confirming the role of this transcription factor for the IL-35 expression^28^. Our results on CD4 + CD25 + Foxp3 + cells agrees with those published and obtained in one patient desensitized to RTX^15^.

Overall, it seems evident that after the initial phases of the DD procedure, this is later redefined during the evolution of the entire process by an increase of regulatory T cells and regulatory cytokines and confirms that the desensitization procedure relevantly impacts the immunologic response, more than has been demonstrated to date.

Although the immunological changes here described shall be confirmed by studying a larger case series, however, surprisingly, they sound very similar between the two patients here described and with another patient desensitized with a third BA (Infliximab)^19^. Since the three patients suffered from different diseases and were treated with different BA, this could suggest a single regulatory mechanism triggered by the DD procedure.

In conclusion DD likely works through two independent mechanisms: (a) the release of mast cell mediators over time; (b) the development of antigen-specific regulation of adaptive response. Both mechanisms are antigen-specific and prevent new adverse reactions from occurring.

## Supplementary Information

Supplementary Information 1.
