# Supplementary material for: Drug-specific Treg cells are induced during desensitization procedure for rituximab and tocilizumab in patients with anaphylaxis
Source: Sci Rep. 2021 Jun 15;11:12558. doi: 10.1038/s41598-021-91851-7 (PMC8206099; doi:10.1038/s41598-021-91851-7)
Supplement: Supplementary file 1 — Supplementary Information 1. [file 41598_2021_91851_MOESM1_ESM.docx]

# Drug-specific Treg cells are induced during desensitization procedure for Rituximab and Tocilizumab in patients with anaphylaxis

# Alessandra Vultaggio ^a^ MD PhD, Francesca Nencini ^a^BSc, Susanna Bormioli ^b^ MD, Elena Silvestri ^c^ MD, Laura Dies ^b^ MD, Emanuele Vivarelli ^a^ MD, Enrico Maggi ^d^ MD, Andrea Matucci ^a^ MD

# ^a^ Immunoallergology Unit, Dept of Medicine and Geriatrics, Careggi University Hospital, Florence, Italy

# ^b^ Centre for Research, Transfer and High Education DENOTHE and Department of Experimental and Clinical Medicine, University of Florence, Florence, Italy

# ^c^ Interdisciplinary Internal Medicine Unit, Neuroskeletal Department and Sense Organs, Careggi University Hospital, Florence, Italy

# ^d^ Translational Immunology Unit, Immunology Area, Pediatric Hospital Bambino Gesù, I.R.C.C.S. Rome. Italy

# AV and FN equally contributed to the paper

# Corresponding author: Prof Enrico Maggi, Translational Immunology Unit, Immunology Area, Pediatric Hospital Bambino Gesù, I.R.C.C.S. Rome Italy, [enrico.maggi@opbg.net](mailto:enrico.maggi@opbg.net)


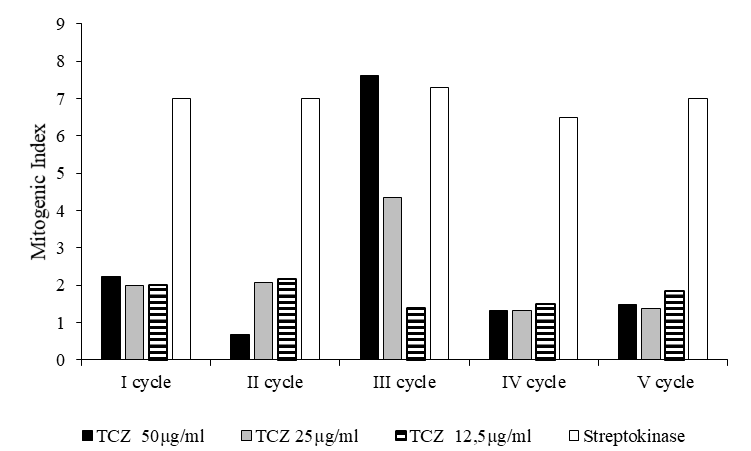


**eFigure 1**. Desensitization effects on PBMC proliferation to 3 TCZ concentrations and streptokinase, evaluated by 3^H^ uptake. *PBMC*, peripheral blood mononuclear cells.

**eTable 1: 16-step desensitization protocol to drug**

| **Steps** | **Solution** | **Rate (ml/h)** | **Time (min)** | **Volume infused (ml)/step** | **Dose administered (mg)/step** |
| --- | --- | --- | --- | --- | --- |
| **DD protocol to Rituximab** | | | | | |
| 1 | 1 | 2.5 | 15 | 0.625 | 0.0016875 |
| 2 | 1 | 5 | 15 | 1.25 | 0.003375 |
| 3 | 1 | 10 | 15 | 2.5 | 0.00675 |
| 4 | 1 | 20 | 15 | 5 | 0.0135 |
| 5 | 2 | 2.5 | 15 | 0.625 | 0.016875 |
| 6 | 2 | 5 | 15 | 1.25 | 0.03375 |
| 7 | 2 | 10 | 15 | 2.5 | 0.0675 |
| 8 | 2 | 20 | 15 | 5 | 0.135 |
| 9 | 3 | 5 | 15 | 1.25 | 0.3375 |
| 10 | 3 | 10 | 15 | 2.5 | 0.675 |
| 11 | 3 | 20 | 15 | 5 | 1.35 |
| 12 | 3 | 40 | 15 | 10 | 2.7 |
| 13 | 4 | 10 | 15 | 2.5 | 6.75 |
| 14 | 4 | 20 | 15 | 5 | 13.5 |
| 15 | 4 | 40 | 15 | 10 | 27 |
| 16 | 4 | 60 | 231 | 231 | 622.4 |
| **DD protocol to Tocilizumab** | | | | | |
| 1 | 1 | 2.5 | 15 | 0.625 | 0.0015 |
| 2 | 1 | 5 | 15 | 1.25 | 0.003 |
| 3 | 1 | 10 | 15 | 2.5 | 0.006 |
| 4 | 1 | 20 | 15 | 5 | 0.012 |
| 5 | 2 | 2.5 | 15 | 0.625 | 0.015 |
| 6 | 2 | 5 | 15 | 1.25 | 0.03 |
| 7 | 2 | 10 | 15 | 2.5 | 0.06 |
| 8 | 2 | 20 | 15 | 5 | 0.12 |
| 9 | 3 | 5 | 15 | 1.25 | 0.3 |
| 10 | 3 | 10 | 15 | 2.5 | 0.6 |
| 11 | 3 | 20 | 15 | 5 | 1.2 |
| 12 | 3 | 40 | 15 | 10 | 2.4 |
| 13 | 4 | 10 | 15 | 2.5 | 6 |
| 14 | 4 | 20 | 15 | 5 | 12 |
| 15 | 4 | 40 | 15 | 10 | 24 |
| 16 | 4 | 60 | 173 | 231 | 553.3 |

**eTable 2: Antibodies and isotype controls for FACS analysis**

| **Antibodies** | **Isotype Controls** |
| --- | --- |
| Human CD3 VioGreen, REA613 | REA Control(s) VioGreen |
| Human CD4 APC-Vio770, REA623 | REA Control(s) APC-Vio770 |
| Human CD25 PE, RE570 | REA Control(s) PE |
| Human Foxp3 APC 3G3 | Mouse IgG1 APC |
